# The Potential of Magnetic Nanoparticles for Diagnosis and Treatment of Cancer Based on Body Magnetic Field and Organ-on-the-Chip

**DOI:** 10.15171/apb.2019.043

**Published:** 2019-08-01

**Authors:** Ali Alirezaie Alavijeh, Mohammad Barati, Meisam Barati, Hussein Abbasi Dehkordi

**Affiliations:** ^1^Department of Clinical Sciences, Faculty of Veterinary Medicine, Shahrekord University, Shahrekord, Iran.; ^2^Department of Applied Chemistry, Faculty of Chemistry, University of Kashan, Kashan, Iran.; ^3^Student Research Committee, Department of Cellular and Molecular Nutrition, Faculty of Nutrition and Food Technology, Shahid Beheshti University of Medical Sciences, Tehran, Iran.

**Keywords:** Body magnetic fields, Cancer, Drug delivery, Magnetic nanoparticles

## Abstract

Cancer is an abnormal cell growth which tends to proliferate in an uncontrolled way and, in some cases, leads to metastasis. If cancer is left untreated, it can immediately cause death. The use of magnetic nanoparticles (MNPs) as a drug delivery system will enable drugs to target tissues and cell types precisely. This study describes usual strategies and consideration for the synthesis of MNPs and incorporates payload drug on MNPs. They have advantages such as visual targeting and delivering which will be discussed in this review. In addition, we considered body magnetic field to make drug delivery process more effective and safer by the application of MNPs and tumor-on-chip.

## Introduction

The development of magnetic nanoparticles (MNPs) is promising for numerous applications. The core-shell nanoparticle is mostly designed for targeted and controlled drug delivery.^1,2^ MNPs can be manipulated through using magnetic fields,^3,4^ and such procedure is categorized as a magnetic resonance imaging (MRI).^5,6^ These particles have relatively low magnetization which has limited their separation in external magnetic field, even if such separation is very important, especially in case of immobilized enzymes collecting.^7,8^ Besides, magnetic field can be changed to achieve thermal treatment.^9,10^ The shell of MNPs is coated with a natural, and synthetic polymer or material copolymer.^1,11,12^ Researchers improve various factors such as temperature, pH, and dual stimuli responsive polymeric nanomaterials such as micelles, vesicles, gels to contribute to cancer treatment.^13,14^ Due to recent researches, silica nanoparticles,^15,16^ iron oxide MNPs, and multi-metallic MNPs, incorporated into dual stimuli responsive polymeric functionalities, have attracted attentions to biomedical applications.^17-23^

Today, anti-cancer drug discovery has mostly been conducted using *in-vitro* and animal models. Animal models for cancer such as mouse and rat models may not attribute to humans accurately, and *in-vitro* models may not be able to simulate tumor microenvironment (TME); thus, they are not appropriate to explore the interactions of cells and organs.^24-27^

Technology of tumor-on-a-chip has recently been introduced as a new approach for cancer research while providing a novel tool which considers microfabrication, biomaterials research, microfluidics, and tissue engineering all together. A tumor-on-a-chip comprises of a microchannel surrounded by a kind hydrogel matrix that is made up of collagen. This system can be used to simulate key aspects of nanoparticle transport such as nanoparticle uptake, diffusion within the extracellular matrix, and extravasation in the tumor.^28-31^

Currently, a number of methods have been put forth for the separation of particles such as acoustic separation, fluorescence, size-based separation, dielectrophoresis, and magnetic control, on tumor-on-a-chip systems. Among these methods, those which are based on magnetic-activated separation are more attractive since they utilize functionalized magnetic particles to capture specific targets through binding and to separate the complex by magnetic manipulation afterwards.^30,32-37^

In addition, to cancer diagnosis and treatment, MNPs can also be used to treat infectious diseases. Yung et al has created an organ-on-chip for blood cleansing using magnetic opsonins targeting *Candida albicans*. The microorganism is, then, cleaned by a micromagnetic separator.^38^

This review describes strategies and considerations of MNPs preparation for drug carrier applications. MNPs have some advantages such as effective targeting and delivering which will be discussed in this review. In addition, we considered body magnetic field that can further drug delivery process more effectively and safely by using MNPs.

## Magnetic field of body organs

Approximately, one Hertz (Hz) frequency is known as a static magnetic field that has different effects on the body. A magnetic field with 0.8T, 22 ms and 1 Hz frequency hampers the growth of S-180 sarcoma in mice. A magnetic field with same properties has been used for patient treatments in their middle and late-stage.^39^ Density and electric field in the tissue and body are expressed in A/m^2^ or mA/m^2^ and V/m or mV/m. During the last years, electric or magnetic fields and current density of human body have been modeled by inducing electric fields at 50 or 60 Hz.^40,41^ Currently, heterogeneous models have been developed with MRI for human body with proper tissue types.^42,43^ Typically, cubic cells (Voxels) are measured for various distinct organs and tissues.^44^ Several organs and tissues have been computed and induced in an electric field.^45,46^ Gap-connected cells have been investigated by long cables model (Table 1).^47^

**Table 1:** Presents electric field induced in several organs and tissues at 60 Hz, 1 µT magnetic field oriented front-to-back^a^

**Tissue/organ**	**Mean**		**99th Percentile**		**Maximum**	
	**50 Hz**	**60 Hz**	**50 Hz**	**60 Hz**	**50 Hz**	**60 Hz**
Liver	13/2		28/2		73/1	
Lung	8/22	21	24/4	49	93/3	86
Blood	5/99	6/9	17/5	23	30/9	83
Uterus	3/81		9/44		17/0	
Prostate		17		36		52
Ovary	2/40		5/30		7/87	
Breast	18/1		31		51/6	

^a^Adopted from Dawson et al.^47^

## Magnetic field of cancer cell

Cancer is still one of the principal causes of death in the developed countries. Conventional therapies for cancer such as surgery, radiation, chemotherapy, and biological therapies have varied disadvantages. The tumor accessibility, the operating risk on vital organs, the cancer cells spreading throughout the body, and the lack of selectivity toward tumor cells are some of these disadvantages. Immunotherapy has been applied for the treatment of small tumors since its efficacy decreases in more advanced stages of cancer. Multimodal therapy has been applied to provide a better chance of survival.^48^

Recent investigation shows the presence of non-erythroid cell lines, stemming from cancer cells of human that show paramagneticproperties.^49^ The cell behavior can be affected with the use of high gradient magnetic filtration according to this property. The inter and intracellular free radicals (O_3_, NO, and NO_2_), molecules (O_2_) and salts (FeCl_3_) can be redistributed using Lorentz force and magnetic gradient.^50,51^

Recent studies have shown that the magnetic and mechanical forces can cause physical interactions that are able to change the cell shapes, functions, and fate change.^52-54^ Cell division can be limited by mechanical stress near the spheroid surface of cancer cells.^55^ The idea of magnetic behavior is enriched in the presence of iron ions and consequently leads to the difference in paramagnetic properties between them and healthy ones. Magnetic radial pressure can make tumor cells paramagnetic and limit tumor growth. Magnetic effects on mouse tumor cell were examined with luminescence, while the cell growth rate dose was not affected. In addition, human fibroblastoma (DMD-A) and human melanoma did not change in growth rate in two cell line culture when a magnetic field with magnetic flux density of 47 T was applied for 72 h. Magnetic fluid hyperthermia (MFH) is another controlled therapy which is non-invasive and supplements chemotherapy. This method can achieve a much higher rate of cancer-cell destruction, either *in vitro* or *in vivo.*^56^

## Magnetic nanoparticles

**Figure 1:**
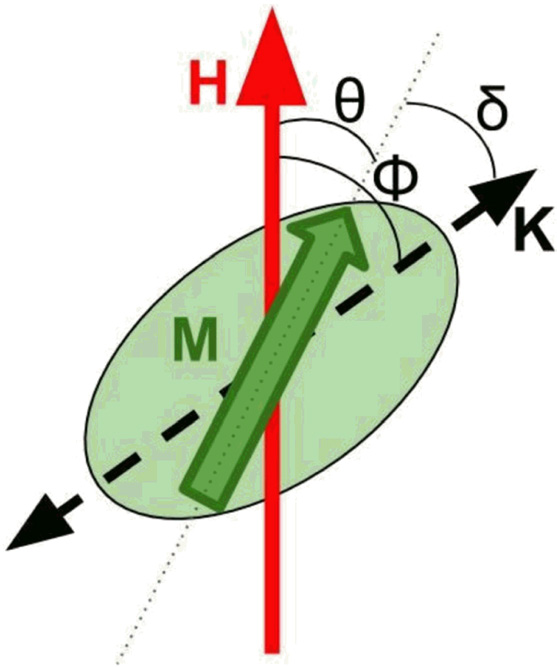
MNP model. The angle h between the magnetization M and the field H, and the angle d between M and the easy axis K, determine the energy.^59^ MNP: magnetic nanoparticles.

The crystal structure, more precisely the orbital electron arrangement in Fe and some other materials creates ferromagnetic species. The iron atomic number is 26, and this electronic configuration has four unpaired electrons in the last orbital that display some associated characteristics and behavior toward other atoms. A single electron has a quantum number of Ms+(1/2) or Ms– (1/2). It is possible for the transformation of ferromagnetic materials to permanent magnet using a strong attraction by magnetic fields.^57^ The metallic nanoparticles are synthesized and modified, utilizing in biomedical science and engineering. The widespread application of them is unavoidable, specifically in biotechnology, magnetic separation, drug delivery, vehicle for gene delivery fields, and mainly in the field of diagnostic imaging; for example, MRI, computerized tomography (CT), positron emission tomography (PET), ultrasound, optical imaging and surface-enhanced Raman scattering (SERS). Iron oxide (Fe_3_O_4_), gold, and silver are different MNPs.^58^ Iron, cobalt, or nickel are used to synthesize metallic MNPs. Oxide of metal MNP scan be protected by the use of coating of silica or gold to prepare core-shell structure.^1^ The volume, shape, composition, and matrix viscosity of the magnets as well as the temperature are the factors which determine magnetic behavior. In a simple and useful model, MNPs with volume of V and saturation magnetization of Ms are considered as species with the shapes of spherical ellipsoids which have a permanent moment m¼MsV. Combination of metallic nanoparticles and MNPs are opening new aspects of application in medicine (Figure 1).^59^

Magnetic hyperthermia nanoparticles have major potentials for clinical application. They are vehicles that are used for cell killing and damaging as well as for increasing the sensitivity to the radiation effects and*/*or certain anticancer drug.^60,61^ Tumors temperature typically fluctuates between 41–46°C (moderate hyperthermia) or *>*46°C (thermo ablation) for a specific duration of time.^61^ Tumor cells usually have higher heat sensitivity over normal cells, at milder temperatures around 43°C, resulting in damage to tumor cells only.^62,63^

Tumors will be warmed by using various techniques such as radio, ultrasound, or infrared waves that can stimulate fatal side effects.^64-67^ There is a relationship between the size distribution and the magnetic properties of the NPs. Hence, very small NPs (∼1–30 nm) do not show sufficient hyperthermia effects and too big ones (*>*200 nm) are not able to cross the endothelial barrier. Treatment with MNP hyperthermia was initially used in 2007 for prostate cancer. When MNPs were applied on metastatic bone tumors, they led to a reduction in the lesion and resulted in new bone formation.^68^

Although there are various methods to synthesize MNPs, it is necessary to develop NPs, which are chemically stable and free from oxidation by oxygen and erosion by acid or base. The MNP is coated by a shell to be protected and to remain stable in harsh chemical situations. There are many advantages in using MNPs with core/shell structure; for instance appropriate dispersion and high stability against oxidation are among them.^69^ There are two types of coating which are divided into organic and inorganic such as silica, carbone and precious metal, or oxides.^70^

**Figure 2:**
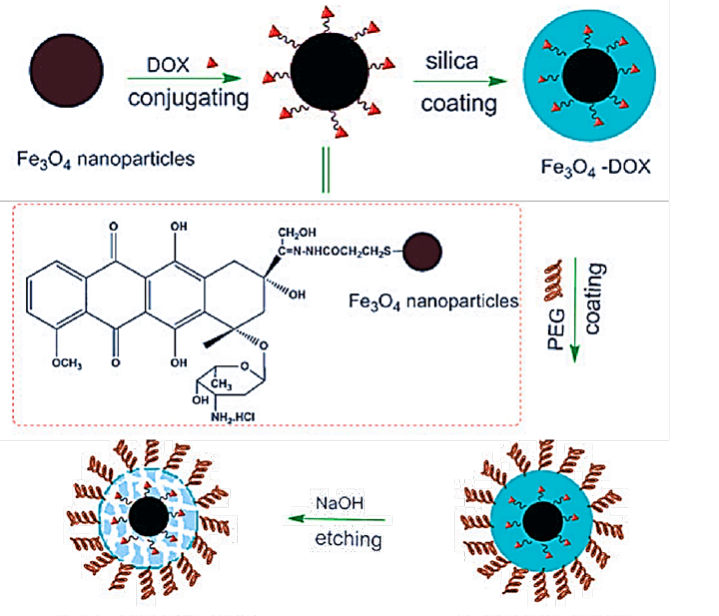
Synthetic scheme for the development of Doxorubicin loaded magnetic drug delivery system (Adopted from Mody et al^99^ ).

**Figure 3:**
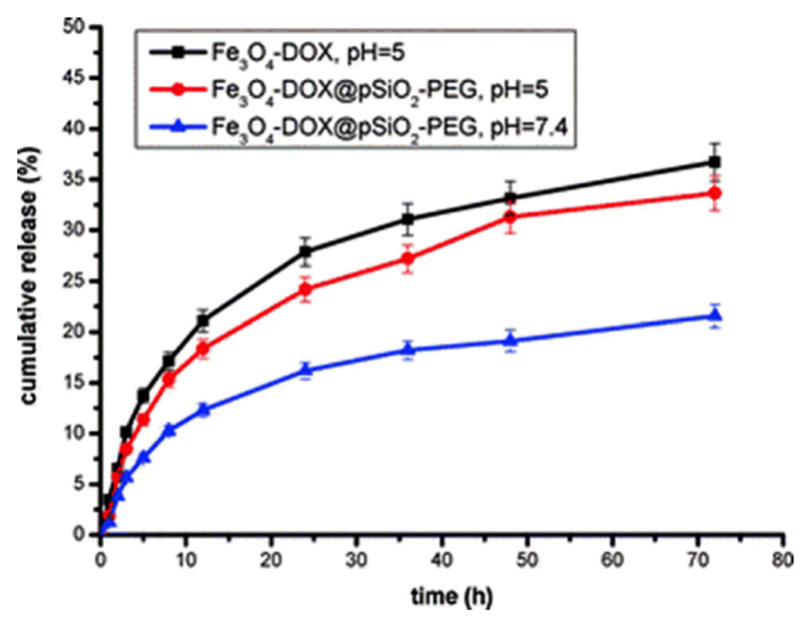
Comparative release profile of Doxorubicin in iron oxide conjugated DOX. Iron oxide conjugated DOX in silica and PEG protected shell (Adopted from Mody et al^99^ ). DOX: doxorubicin; PEG: polyethylene glycol.

The physical absorption and chemical bonding have been used for loading of different molecules on organic and inorganic bases: the tumor-recognition moieties,^71,72^ cell-penetrating peptides for MRI applications,^73,74^ as well as enzymes,^75^ genes,^76-80^ growth factors,^81,82^ radionucleotides,^83,84^ drugs,^85,86^ tamoxifen,^87^ cefradine,^88^ ammonium glycyrrhizinatezz,^89^ fludarabine,^90^ cisplatin and gemcitabine,^91^ amethopterin,^88,92^ mitomycin,^93^ paclitaxel,^94,95^ diclofenac sodium,^96,97^ and Adriamycin^98^ for drug delivery applications (Figures 2 and 3).^99^

The first polymer MNPs and micro particles were first used in the 1970s. This type of coating protects the shield from the surrounding environment and works when coupled with carboxyl groups, carbodi-imide, biotin, avidin, and other molecules. The carrier of MNPs is made up of different structural configuration of magnetic particle core such as Fe_3_O_4_ or Fe_2_O_3_.^100^

## Function and application of MNPs

The MRI agents, based on an oral contrast type of large iron oxide particles for MRI of bowel/GI tract, were the first ones of these agents.^101^ Abdoscan^®^ (Ferristene) is another oral iron-based negative MRI which has been approved in Europe. The size of these MNPs is 50 nm which have been coated with polystyrene to obtain 3.5μm particles.^102^ These MNPs are mainly used to take pictures from liver*/*spleen*/*lymph nodes in patients with pelvic, prostate, bladder, or breast cancer, and this process is briefly elaborated here. Relatively, iron oxide MNPs of large size are used for liver*/*spleen imaging such as AMI-25 (Feridex^®^*/*Endorem^TM^, DH = 80–200 nm) and SHU 555A (i.e., Resovist^®^*/*Cliavist^TM^, DH = 60 nm).^103,104^ Iron oxide of smaller particle size is considered for lymph node*/*bone marrow*/*carotid atherosclerotic plaques imaging (AMI-227 [i.e., Sinerem^®^*/*Combidex^TM^, DH = 20–40 nm]).^105,106^

The idea for using magnetic particles as drug carriers for cancer therapy has been utilized in 1960.^107^ The cytotoxic activity of iron oxide MNPs is insignificant when administered at Fe concentrations up to 100 μg*/*mL,^108^ and even up to 8 mg Fe*/*mL in formulations such as ferumoxytol.^109^ The normal blood iron concentration and clinical doses of such MNPs in humans are about 33 mg Fe/kg and 0.56-8 mg Fe/kg patient body weight which is incomparable.^110^

In addition to afore mentioned point, MNPs potentially can play a crucial role in the new cancer treatment approaches. Among the methods of diagnosis and treatment of cancer, magnetic-activated separation is more attractive for researchers since this method utilizes functionalized magnetic particles to capture specific targets through binding and separates the complex by magnetic manipulation afterwards. The separation relies on the chemical bonds interaction and, hence, specific and selective separations of the particles are allowed.^32-37^ , With this approach, MNPs can also be used to treat infectious diseases. In a study, the blood cleansing from *Candida albicans* was done using magnetic particles.^38^

## Drug delivery

Any agent used in drug delivery is called a drug carrier which serves to enhance the safety, selectivity, and effectiveness of drug administration. Generally, Drug carriers are used for controlled release of a therapeutic agent into circulation. Such release can be caused either by a typical diffusion of drug (slow release) or by a triggered drug deliverance to a specific target by stimuli such as pH, heat, and light. The more popular drug carriers include polymeric micelles, liposomes, microspheres, and nanoparticles.^111,112^

Nanoparticle carriers have considerable potential for cancer diagnosis and treatment. The most important technological benefits of NPs are high carrier capacity, incorporation of both hydrophobic and hydrophilic drugs, and high stability and feasibility of different routes of application including oral administration. The mentioned properties of NPs improve drug bioavailability and dosing frequency reduction, and in some disease may resolve the problem of nonadherence to a therapy.^113-116^ MNPs are a class of NPs which can be manipulated by magnetic fields.^117^

The nanoscience technology is a promising way to detect disease and control drug delivery.^69^ MNPs are very beneficial due to their unique properties of biological interaction which makes them a suitable agent for MRI. Examples of such unique proprieties of MNPs are magnetic moment, non-fouling surface in detection, diagnosis, and treatment of malignant tumors as well as cardiovascular and neurological disease.^1^ MNPs are carriers which are able to deliver drug in a particular area in the body. They can be mixed with drugs and injected to the specific area by an intravenous or intra-arterial injection. Reaching the site, the drug is released from the carrier due to enzymatic activity or a change in pH, osmolality, or temperature. This technique improves the efficacy of drug delivery, reduces the systemic distribution of cytotoxic drugs, and results in an efficient treatment at lower doses.^118^ The “tumor-on-a-chip platforms for NP-transport and testing” section will elaborate more on new MNPs implications.

## Magnetic drug targeting

Magnetic drug targeting is an appropriate method for delivering drugs to diseased area which may need a minimal invasive procedure.^119-121^ The MNPs change the structure of the thermos-sensitive material by generating heat. In addition, the drug is released by the carrier at the tumor site and increases the effectiveness of the therapy.^61^ Under extensive investigation, prostate and brain tumors have been diagnosed by magnetic hyperthermia in humans.^122^

The ideal size of NPs for cancer therapy due to their fast tumor penetration is approximately 12 nm. It has also been shown that 50 nm nano-conjugates can be close to the optimal size for overall tumor tissue accumulation and retention compared with those smaller than 20 nm which are rapidly cleared from the bloodstream and with those larger than 200 nm which have limited tumor tissue penetration.^123,124^

A common occurrence in NPs is the removal of size dependence in the human vascular system. Which depends on morphological pore sizes, involved in diffusive permeability of the capillary vessels. There are some examples of morphological pore size in our body.^125^ The maximum size of NPs is permitting penetration through cell membranes (∼1 μm). The upper size limit for particles with rigid structures flowing in veins is 5 µm. Due to the fact that the size of typical NPs is below the narrowest capillaries, basically there is no limitation concerning delivery procedure. As it was previously mentioned, the most important limitation lies in an increase in the circulation time and the clearance from the body. Because of negative charge of the luminal surface of vascular endothelial layer, the positively charged NPs can rapidly react with endothelial cells by the formation of non-specific bonds, and a reduction in blood circulation.^126^ On the other hand, NPs with highly negative charges are capable of being trapped by some macrophages and organs. It is difficult for neutrally charged MNPs to be sterically stabilized. Hence, for longer blood circulation, it is necessary for MNPs to have a negative or positive surface charge for improved targeting.^127^

To demonstrate optimal capacity to incorporate chemotherapeutic drugs and to be effective delivery vehicles, the therapeutic MNPs should have average sizes between 10 and 100 nm with a proper coating, ζ-potential values between -15 and+15 mV, and an elongated to spherical shape. However, the preparation of such stable colloidal MNPs which are highly controllable for delivering the drugs to targets is still debatable.^128^

## Biotransformation of MNPs

**Figure 4:**
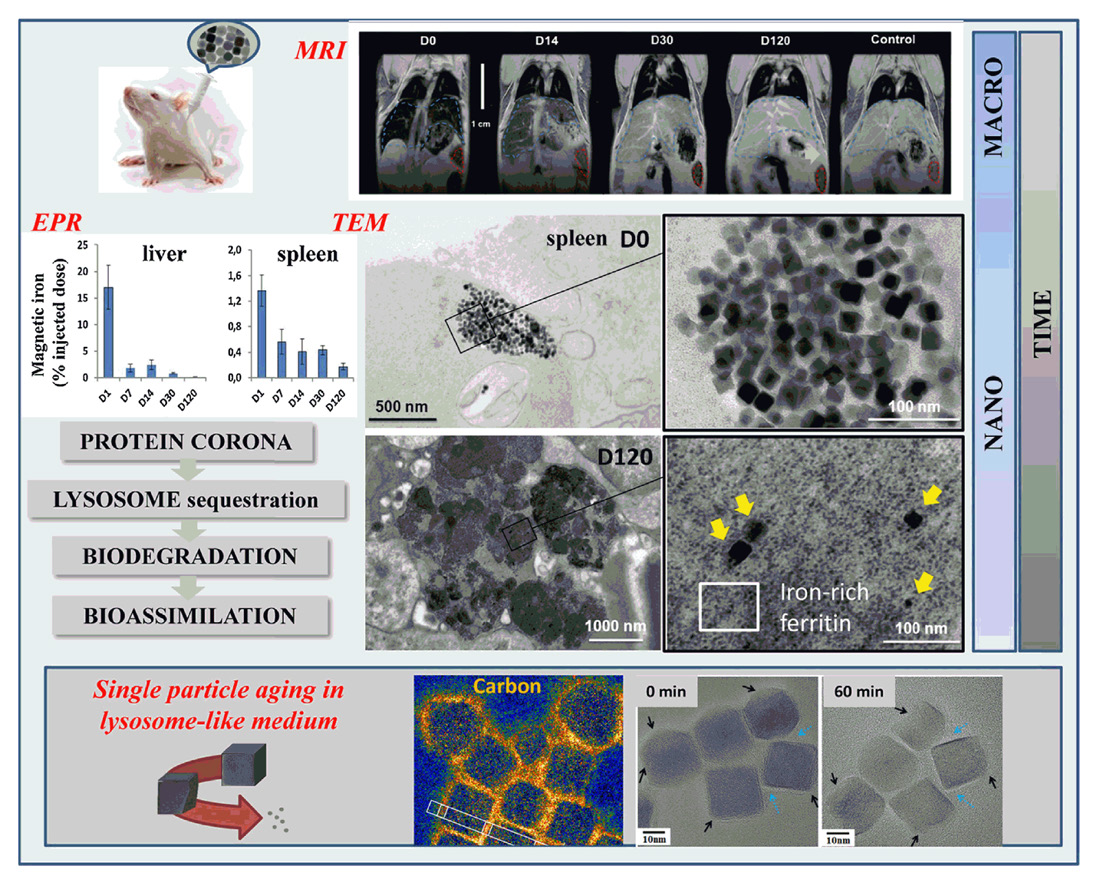
Multiscale follow-up of iron oxide nanocubes over time, using in vivo MRI in mice, ex vivo EPR quantification in organs, TEM observations of intracellular distribution and morphological biotransformations.^136^ MRI: magnetic resonance imaging; EPR: electron paramagnetic resonance; TEM: transmission electron microscopy.

Recent investigations have expressed that the identity and properties of NPs can be changed with biological interactions.^129^ The molecules within the biological fluids reshape NPs’ surface and such change leads into particle aggregation enzymatic attack.^130,131^ NPs remodeling may be effective in regulation of transportation in physiological media, cellular internalization, and potential toxicity. The MNPs degradation may produce harmful by-products which have unexpected biological effects. On the other hand, the saturation of lysosomal compartments may occur with non-degradable NPs accumulation.^132,133^ A suitable external magnetic field can appropriately separate the magnetic NPs from blood as a complex biological media. Importantly, the corona varies over time depends on the stage of cell process.^134^ The observations show that these particles reach liver and spleen. The coating-dependent elimination of super para- magnetic iron from these organs seems to take months. Paramagnetic iron concentrates in liver and spleen and, afterwards, transforms into iron oxide phases with no magnetic properties. Particles coated by amphiphilic polymers are more persistent than polyethylene glycol of hydrophilic chains and they can be observed *in vivo* in liver and spleen one year after injection (Figure 4).^135,136^

## Tumor-on-a-chip platforms for NP-transport and testing

**Figure 5:**
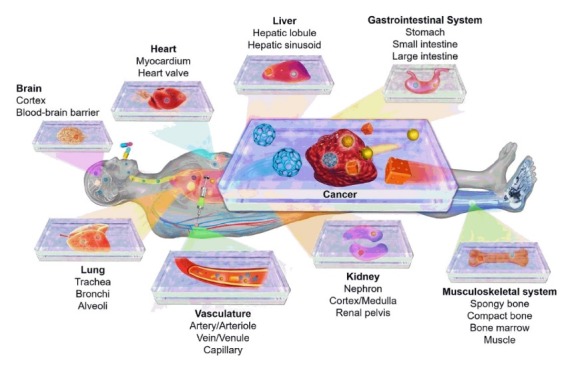
Organ-on-a-chip and disease-on-a-chip platforms for modeling human physiology and pathophysiology.^140^

Tumor-on-a-chip devices provide viable options for promoting the efficacy of cancer therapy.^137^ Today, the study, identification, and treatment of cancer tumor cells circulating (CTCs) are developed by the tumor-on-a-chip device .There are some drawbacks in conventional therapies such as low drug specificity, poor water solubility, lower therapeutic efficiency, and drug resistance which can be eliminated through the utilization of NP-based targeted drug delivery systems (Figure 5).^138-140^

Despite the comprehensive research in cancer biology, TME is not well known. As it was mentioned before, the disease is the leading cause of mortality in the developed countries. Hence, it seems the understanding of TME can have a pivotal role in cancer diagnosis and treatment. Technology of organs-on-chips provides effective approaches to have a deep vision on TME.^128,141-143^

Today, anti-cancer drug discovery has mostly been conducted using *in-vitro* and animal models. Cancer animal models can provide vital information about responses to anti-cancer drugs. Although the models could be low cost, the wide variations among the animals used make it difficult to obtain relevant statistics. Furthermore, animal models for cancer such as mouse and rat models may not attribute to humans accurately.^144^ Alongside *in-vivo* studies, different types of cell culture models have been used for cancer research.^26,27^ Such *in-vitro* studies may co-culture multiple cell types with patient derived cells in a matrix. Although* in-vitro* studies are costly and repeatable, the models may not be able to simulate TME; Therefore, they are not appropriate to explore the interaction of cells and organs.^24,25^

Technology of tumor-on-a-chip has recently been introduced as a new approach for cancer research while providing a novel tool which contains microfabrication, biomaterials research, microfluidics, and tissue engineering all together.^28^ A tumor-on-chip system is comprised of a microfluidic chip which has nutrients, waste removal functions, tissue culture, and small molecule supply. Tumor can grow on the device with a complex structure consisting of blood vessels, tumor cells, and stromal cells three-dimensionally.^28-31^

As it was mentioned before, a tumor-on-a-chip is comprised of a microchannel surrounded by a kind hydrogel matrix which make up of collagen. This system can be used to simulate key aspects of nanoparticle transport such as nanoparticle uptake, diffusion within the extracellular matrix, and extravasation in the tumor. This tumor platform can be explored for developing novel nanoparticles with some focus on emphasized optimizing features such as functionalization method, size, and shape. Consequently, the system can yield the most appropriate nanoparticles with characteristics which facilitate transport within the TME, resulting in more effective cancer treatments with nanoparticles.^145-147^

Currently, a number of methods have been put forth for the separation of particles such as acoustic separation, fluorescence, size-based separation, dielectrophoresis, and magnetic control on tumor-on-a-chip systems. Among these methods, those which are based on magnetic-activated separation are more attractive since they utilize functionalized magnetic particles to capture specific targets through binding and, thus, to separate the complex by magnetic manipulation. The separation relies on the chemical bonds interaction due to which specific and selective separation of the particles can be allowed.^30,32-37^

In addition to cancer diagnosis and treatment, MNPs can also be used to treat infectious diseases. Spleen plays a crucial role in filtering the circulating fluid and is important to body’s immune system. It forms immune responses against certain microorganisms such as *Candida albicans*. Yung et al created an organ-on-chip for blood cleansing using magnetic opsonins targeting *C. albicans*. The microorganism is, then, cleaned by a micromagnetic separator.^38^

Recent studies have attempted to clarify MNPs behavior in response to magnetic fields in 3D microfluidic models. Benhal et al have designed a sample test on-chip--and-image tracking system to assess the movement of MNPs in response to an applied magnetic field. The authors have been able to evaluate the motion of different MNPs ranging from 10 nm to 100 µm in diameter size. All of the particles seem to have displayed consistent trends: i.e., larger MNPs move faster; the particles at higher concentrations make longer needle like aggregates and move faster,; both speed and chain increase with time while yielding a final speed proportional to the square of particle diameter.^148^ In a similar study, Geczy et al have revealed that the resulting particles, through the encapsulation of MNPs, were monosized, highly spherical, and exhibited superparamagnetic properties. The particles’ size regime and their magnetic responses have demonstrated potentials for in-vivo intravenous applications of magnetic targeting with maximum magnetic response without blocking an organ’s capillaries.^149^

## MNPs as a theranostic agent

Theranostic is defined as the combination of diagnostic and therapeutic agents within a single platform.^150^ Table 2 summarized the studies used theranostic multimodal MNPs for diagnosis and treatment of chronic disease especially cancer. The conventional methods for diagnosis and treatment of cancer are biopsy and chemotherapy -- which is very invasive and time-consuming in comparison. Besides, there are several limitations for chemotherapy to achieve desirable results. The entire tumor removal is not applicable by chemotherapy. Therefore, the satisfactory diagnosis and treatment of the metastatic tumor remain a challenge. Such challenge encourages scientists to research on NPs for the detection of and the cure for cancer by paclitaxel and Nano-formulated anthracyclines. Specific drugs and ligands have been conjugated on the NPs surfaces which interact with proliferating cells of tumor leading into accumulation at the tumor. The targeting system provides low chemo-resistance and cytotoxicity and induces the expression of apoptotic genes.^151,152^

**Table 2:** The studies that used theranostic multimodal MNPs for diagnosis and treatment of chronic diseases

**Method of imaging**	**Nanoparticle**	**Disease**	**Therapeutic agent**	**Target**	**Ref.**
Optical	PLGA	Cancer	Camptothecin	Folate receptor	^153^
Optical	Poly(isobutylene-alt-maleic anhydride)	Cancer	Paclitaxel	-	^154^
NIRF	PEG-hyaluronic acid	Cancer	Irinotecan	Hyaluronic acid	^155^
Raman spectroscopy	Gold	Cancer	Doxorubicin	-	^156^
MRI	SPION	Allograft rejection	DGKa-pDNA	CD3 antibody	^157^
MRI	SPIO	Immune response detection	-	-	^158^
Ultrasound	Hyaluronic acid encapsulated with MnO2	Cancer	Indocyanine	Hyaluronic acid	^159^
Ultrasound	Mesoporous silica	Cardiac stem cell therapy	IGF	-	^160^
PET	T7 phage nanoparticle	Cancer	-	RGD	^161^
CT	Glycol-chitosan-coated gold	Cerebrovascular thrombi detection	tPA	Fibrin-binding peptide	^162^

CD: cluster of differentiation; CT: computerized tomography; DGKa: diacylglycerol kinase alpha; IGF: insulin-like growth factor; MNPs: magnetic nanoparticles; MRI: magnetic resonance imaging; NIRF: near infrared fluorescence; PET: positron emission tomography; PEG: polyethylene glycol; PLGA: poly lactic-co-glycolic acid; RGD: arginylglycylaspartic acid; SPIO: superparamagnetic iron oxide; SPION: superparamagnetic iron oxide nanoparticles; tPA: tissue plasminogen activator.

It is believed that MNPs are not toxic at low concentration, and their removal from the biological fluid can be allowed for by metabolic cascades.^151^ The factors which determine the biocompatibility and toxicity of MNPs are the existence of the magnetic components such as cobalt, iron, nickel, magnetite, and the final size, core and coating of the particles. Magnetite or its oxidized form is the most commonly employed NPs for biomedical applications. Highly magnetic compounds are toxic; hence, the compounds are of little interest. However, encapsulation enhances dispersibility, chemical stability, and declines toxicity. The major benefit of using NPs of sizes smaller than 100 nm is their enhanced tissular diffusion, lower sedimentation rates, and higher effective surface areas. Another benefit of using this type of NPs is a meaningful decline in the interactions of the magnetic dipole-dipole due to the compounds scale of r6.^163-165^ Table 3 summarizes the studies which have used organ-on-a-chip or 2D microfluidic models for drug delivery.

**Table 3:** Organ-on-a-chip and 2D microfluidic models for drug delivery

**Cells**	**Culture**	**Nanoparticle**	**System**	**Brief summary of the study**	**Ref.**
A549	2D dish culture	Cerium oxide NPs	-	Oxidative stress and cytotoxicity effects of CeO2 NPs on A549 cells	^166^
Human bronchoalveolar carcinoma	2D dish culture	Silica NPs	-	The effect of Silica NPs size on toxicity in bronchoalveolar carcinoma	^167^
LCC6/Her2	Droplet based microfluidics	DOX loaded CaCO3-NPs	In this system, the alginate beads were trapped in micro-sieve structures for cell culture in a continuous perfusion system. The environment permitted cell proliferation and the formation of multicellular spheroids.	Dose dependent cytotoxic effects of DOX loaded CaCO2-NPs	^168^
Human endothelial cell	2D microfluidic channel	MSN	A simple microfluidic platform with precisely controlled shear stress conditions	Shear stress and endothelial cytotoxicity	^169^
MDA-MB-435	Organ-on-a-chip (spheroid formation)	Gold NPs	A tumor-on-a-chip system where incorporation of tumor-like spheroids into a microfluidic channel permits real-time analysis of NP accumulation at physiological flow conditions.	Developing a tumor-on-a-chip system to study the transport of gold NPs through a 3D tissue environment and characterizing NPs within a tumor tissue.	^170^
Hy926 and human platelet	2D microfluidic channel	FMS-NPs	The impact of sub-50 nm diameter mesoporous silica nanoparticles on platelet function is investigated using a microfluidic platform to model blood vessel characteristics	The effect of FMS-NPs on platelet aggregation	^171^
HUVEC	2D microfluidic channel	Lipid-polymer NPs	Endothelialized microchip with controllable permeability can be used to probe nanoparticle translocation across an endothelial cell layer.	Microfluidic model of atherosclerosis to assessment of NPs endothelial translocation	^172^
MCF-7 and MVECs	Organ-on-a-chip (Pseudo 3D)	Fluorescent NPs	This model, consists of 3-dimensional microfluidic channels where tumor cells and endothelial cells are cultured within extracellular matrix under perfusion of interstitial fluid	Transport of NPs within the tumor	^173^
Caco-2, TH29-MTX and HepG2/C3A	2D microfluidic channel	carboxylated polystyrene NPs	To construct this system, we combined in vitro models of the human intestinal epithelium, represented by a co-culture of Caco-2, TH29-MTX, and HepG2/C3A cells, within one microfluidic device.	Detection of liver injury using GI tract-liver-other tissue system	^174^
HUVEC	2D microfluidic channel	Gold NPs	The tests performed in the microfluidic device were also run in multiwells, where no flow is present	Flow dependent cytotoxicity effects of gold NPs on endothelial	^175^
MCF-7, MDA-MB-231, and SUM-159PT	Organ-on-a-chip (3D gel pattering)	Dox-HANP	Three types of human breast cancer cell lines including MCF-7, MDA-MB-231, and SUM-159PT were cultured on a 3D platform, and their drug response and resistance to doxorubicin were characterized	The effects of NPs mediated drug delivery	^176^
HUVEC	2D microfluidic channel	Gold NPs	A microfluidic device to observe how HUVEC viability changes when subject to a continuous flow of culture medium.	The effect of shear stress and gold NPs size on endothelial cytotoxicity	^177^
HUVEC	Organ-on-a-chip	HDL mimetic NPs	A micro-engineered three-dimensional vascular system	The effect of HDL mimetic NPs on endothelial cells	^178^

CaCO3: calcium carbonate; CeO2: cerium oxide; DOX: doxorubicin; FMS: fluorescent mesoporous silica; MSN: mesoporous silica nanoparticle; HDL: high density lipoprotein; HANP: hyaluronic acid nanoparticle; gH625: membranotropic peptide; NPs: nanoparticles.

## MNPs toxicity

There are some advantages in using MNPs for cancer diagnosis and treatment. On the contrary, due to some toxicity associated with the use of MNPs many restrictions have been identified in their application. Potential toxicity and some other features of MNPs are influenced by surface coatings of them. It has been illustrated in the research that there is positive relation in the size of the MNPs and accumulation. Therefore, if the size and surface coatings of MNPs have been controlled, it can reduce toxicity and improve magnetic behaviors. Currently, several magnetic materials with a broad spectrum of magnetic attributes are available. The high toxicity of some materials such as cobalt and chromium make them useless in biomedical applications. Such threats can be removed by a non- toxic coating which has high mechanical strength. Many studies have been conducted to develop different techniques for using Gadolinium NPs in medical imaging. Although the utilization of this compound for patients with renal failure is still controversial, Iron oxide NPs can be a good alternative for such patients. Due to the importance of the size in nanoparticles, the proportion of elements at the time when iron oxide is used as a contrast agent may be problematic, because the reason may lie in the fact that the size of the nanoparticles counts significantly. However, other types of paramagnetic and superparamagnetic NPs have been developed to overcome such drawbacks.^163-165^

## Conclusion

NPs are self-assembled molecules which behave in a controlled manner. In some biomedical applications we need to use core shell MNPs to insure the biocompatibility and stability of biomolecules. In some biomedical applications we need to use core shell MNPs to insure the biocompatibility and stability of biomolecules. In some biomedical applications we need to use core shell MNPs to insure the biocompatibility and stability of biomolecules. In some biomedical applications we need to use core shell MNPs to insure the biocompatibility and stability of biomolecules. Nasopharyngeal carcinoma (NPC) was treated with cisplatin which was loaded on folate MNPs. Accordingly, they were targeted and accumulated in NPC cells efficiently. MNPs have some benefits for targeted drug delivery under image guidance which have been discussed in this review. We considered body magnetic fields to improve the efficiency and safety of drug delivery by using MNPs. Novel technologies such as tumor-on-a-chip platforms can offer other benefits along with in-vitro model which can be enjoyed in order to overcome and treat cancers. The combination of pharmacodynamics and pharmacokinetics NPs allow us to optimize the effectiveness of such treatment.

## Ethical Issues

This article does not contain any studies with human and animal subjects performed by any of the authors.

## Conflict of Interest

The authors declare that they have no conflict of interest.

## Acknowledgments

The authors are grateful to University of Kashan for supporting this work by Grant No. 785112.
